# Septic shock, acute renal and liver failure following unsafe abortion using bitter leaves and wandering jew in northern Uganda: A case series

**DOI:** 10.1002/ccr3.5975

**Published:** 2022-06-27

**Authors:** Gasthony Alobo, Cristina Reverzani, Violah Nahurira, Laura Sarno

**Affiliations:** ^1^ Department of Obstetrics and Gynaecology Lira University Lira Uganda; ^2^ Department of Obstetrics and Gynaecology St. Mary's Hospital Lacor Gulu Uganda; ^3^ Department of Neurosciences, Reproductive Science and Dentistry University of Naples Federico II Naples Italy

**Keywords:** case series, herbal medicines, herbs, northern Uganda, unsafe abortion

## Abstract

Unsafe abortion is a major problem in Uganda, being one of the leading causes of maternal morbidity and mortality. Abortions are performed mostly under unsafe conditions, by people without medical training. In rural areas in northern Uganda, women often resort to traditional providers, who use local herbs as abortion remedies, usually with adverse outcomes. Little is known about the biological properties of these herbs and their toxicity profile. Here, we present the case series of two women, of 31 and 24 years of age, who underwent unsafe abortion for unintended pregnancy by using herbal medicines, that is, *Commelina Africana* (wandering jew) and *Vernonia amygdalina* (bitter leaf), respectively. While the first case resulted in uterine necrosis and pelvic peritonitis, which required multiple surgical interventions and the use of reserve antibiotics, the second case resulted in liver and renal failure that led to the death of the patient. This case series describes the unusual severe toxicity of two herbal medicines that are frequently used to induce abortion in northern Uganda. It highlights possible associations of *Commelina Africana* (wandering jew) with uterine necrosis complicated by sepsis, and of *Vernonia amygdalina* (bitter leaf) with acute liver and renal failure.

## 1 BACKGROUND

Worldwide, the leading causes of maternal morbidity and mortality are direct obstetric complications and include hemorrhage, sepsis, hypertensive disorders, and abortion; the latter is responsible for 7.9% of maternal deaths globally and 9.6% in sub‐Saharan Africa (SSA).^1^ The World Health Organization defines unsafe abortion as a procedure for terminating a pregnancy performed by people lacking the necessary skills or in an environment, not in conformity with minimal medical standards, or both.^2^

In Uganda, although the official data of the Ministry of Health indicate that abortion accounts for 5% of maternal deaths,^3^ the mortality due to abortion complications could be higher due to restrictive laws. It is estimated that 297,000 unsafe abortions are performed yearly, with an abortion rate of 54 per 1000 women aged 15–49. Nearly 85,000 women have been treated annually for complications of unsafe that include incomplete abortion, sepsis, and uterine perforations^4^ making it the main gynecologic cause of admission, leading to about 1200 deaths annually.^5^

The abortion rate is even higher in northern Uganda (70 per 1000 women aged 15–49 years),^4^ a predominantly rural region that is still recovering from a 20‐year conflict that left many people displaced from their homes and economically disadvantaged. One of the factors contributing to the high number of abortion complications is the unmet need for contraception, which is still very high (28%). Contraceptive use in Uganda remains very low; only 17% of all women of reproductive age, and 18% of married women, utilize modern contraception.^6^ This has resulted in a high total fertility rate (TFR) of 5.4.^7^ It is estimated that almost half of the pregnancies in Uganda are unintended, with 15% of the total pregnancies ending in unsafe abortion and constituting nearly one‐third of the maternal deaths among the country's young people.^8^ The situation is even more dramatic among rural, poor, and less educated women, such as in the northern region.

Legal provisions for abortion in Uganda are very restrictive. Art. 22(2) of the Constitution of Uganda (1995) quotes: “No person has the right to terminate the life of an unborn child except as may be authorized by law.” The Penal Code Act (Cap. 120), as amended through the Penal Code Amendment Act (No. 8 of 2007), Section 205, allows only abortion if “carried out in good faith to preserve the mother's life.” Therefore, safe “legal” abortion is not available and accessible, except for rare cases where it is the only way to save a woman's life, and many healthcare providers prefer not to offer safe abortion to women who need it.^9^ It is known that highly restrictive laws do not reduce abortion rates, but rather increase the likelihood of unsafe abortion.^10^ Consequently, in a country like Uganda where abortion is highly stigmatized,^11^, ^12^ many women seek illegal abortion with unsafe traditional methods, offered by non‐medical providers under unsafe conditions. While in urban areas the use of prostaglandin analogs is the most common method of unsafe abortion, in rural areas women resort to the intra‐vaginal use of sharp objects (safety pins, nails, knives, or sticks), to the ingestion of caustic substances (detergents or bleach) and more often to the use of local herbs, such as *Commelina Africana* and *Vernonia amygdalina*, which are readily available in the countryside.^13^

In this small case series, we described two cases of unsafe abortions induced with very common herbs in northern Uganda, which ended up with severe complications and, in one case, led to the death of the woman.

## 2 CASE PRESENTATION

### 2.1 Case 1

N.A., a 31‐year‐old female, para 2, abortus 1, at 5 weeks of amenorrhea and HIV negative, was admitted to our Gynecology ward via the Emergency Department. The patient presented with a 2‐week history of lower abdominal pain, 3 days of vomiting, and 2 days of loose stools. She did not report any bleeding or discharge per vagina; the micturition habit was normal. She reported not being pregnant in recent months and that the symptoms started spontaneously, without any identifiable cause, and worsened progressively.

At admission, the patient was conscious, with Glasgow Coma Scale (GCS) of 15/15, but pale and in obvious discomfort; her blood pressure (BP) was 96/56 mmHg, pulse rate (PR) was 112 beats per minute, and temperature was 38.7°C. Abdominal examination revealed severe distension, generalized tenderness, and guarding; bowel sounds were present. The vaginal examination demonstrated no active bleeding but moderate foul‐smelling discharge; the cervical os was closed. At the bimanual examination, the uterus was bulky, with severe cervical motion tenderness. The physical examination of the other systems was unremarkable.

Her blood investigations showed hemoglobin (Hb) of 6.5 gm/dl, white cell count of 32.3 × 10^9^/L, platelets of 105 × 10^9^/L. The urine human chorionic gonadotropin (hCG) quick test was positive. A pelvic ultrasound (US) scan showed an empty and bulky uterus, normal‐looking ovaries, and the presence of an echogenic pelvic mass; a diagnosis of a pelvic abscess was made and an exploratory laparotomy planned, after resuscitation of the patient. During the exploratory laparotomy, the uterus was severely necrotic and there was pus collection of 2 L in the paracolic gutters and the sub‐diaphragmatic recess.

During the postoperative period, antibiotic therapy with intravenous first‐line broad‐spectrum antibiotics was performed, but the patient remained febrile and started discharging pus from the laparotomy wound on the 5th postoperative day. A wound swab culture was performed, revealing a multi‐resistant *Escherichia coli*, sensitive to Gentamicin and Chloramphenicol. Therapy with a combination of these antibiotics did not show any improvement in the following days and, on the 15th postoperative day, the incision site started gaping, with copious pus discharge.

A further pelvic ultrasound scan showed an echo‐complex fluid collection in the pouch of Douglas, with a volume of around 265 ml; the findings were in line with a pelvic abscess. A repeat laparotomy confirmed the diagnostic hypothesis and an extensive pelvic lavage was done. The patient was treated postoperatively with a reserve antibiotic (intravenous Meropenem) and after 10 days was discharged home in good general conditions, on oral antibiotics. At discharge from the hospital, the patient reported having induced the abortion with a local herb, known as “wandering jew,” 3 weeks before admission, by inserting some pieces of the stem of the plant in the vagina; a few days after the expulsion of the fetus, she started developing lower abdominal pain and abdominal distension, associated with per vagina foul‐smelling discharge.

### 2.2 Case 2

A.E., a 24‐year‐old female, gravida 4, para 3, at unknown weeks of amenorrhea, was admitted to our Gynecology ward with a history of fever and chills, general body weakness, tea‐colored urine, and cramping lower abdominal pain, radiating to the waist, that started spontaneously and increased progressively in intensity. There was no history of vomiting or nausea, vaginal bleeding, or discharge; the patient reported normal micturition and bowel habits. The history of complaints of other systems was unremarkable.

The patient, who was married with three living children, did not attend antenatal care for the current pregnancy; obstetric and gynecological histories were unremarkable. There was no history of chronic illnesses, while the HIV status was unknown. The patient denied the use of herbal medicine or any other medication in this pregnancy.

On physical examination, she was sick‐looking, with moderate pallor severe jaundice with dry mucous membranes, dry skin, and no edema. Her BP was 93/61 mmHg, with PR of 94 beats per minute and a temperature of 36.8°C.

Abdominal examination revealed a normal fullness, with a moderate tenderness in the hypogastric region; no organomegaly or masses were palpable. On per vagina examination, vulva and vagina were normal, the cervical os was closed and there was no active bleeding or discharge. The physical examination of the other systems was unremarkable.

Her investigations showed a positive urine hCG quick test, while the blood smear did not evidence any malaria parasites. The complete blood count showed a white cell count of 28.1 × 10^9^/L, with granulocytes of 20.5 × 10^9^/L, Hb of 11.7 g/dl, and red blood cells of 3.66 × 10^12^/L, platelets of 270 × 10^9^/L, and negative hepatitis B surface antigen test. Liver function: Alanine transferase of 1796 U/L (0–40), Aspartate transferase of 1628 U/L (0–37), Alkaline phosphatase of 159 U/L (98–279), and albumin of 2.5 g/dl (3.8–5.1). Renal function test: urea of 82 mg/dl (10–55), creatinine of 7.1 mg/dL (0–1.3), and urea/creatinine ratio of 11.54. Serum electrolytes: sodium of 128.2 mmol/L (134–146), potassium of 4.6 mmol/L (3.5–5.5), and chloride of 105.9 mmol/L (98–108).

A pelvic ultrasound scan showed a gravid uterus with an intrauterine fetus in cephalic presentation, but no cardiac activity was present; the amniotic fluid was reduced and the internal cervical os was open. Moreover, the left maternal kidney was mildly enlarged and echogenic, with normal margins. The findings of the ultrasound scan were in line with a missed abortion at 14 weeks of gestation and maternal left nephropathy. Our impression was a para 3, abortus 1, with missed abortion at 14 weeks of gestation, acute hepatitis, and nephropathy. The patient was stabilized and managed conservatively, without relevant improvement.

On the 2nd day of hospital stay, there was a new complaint of bleeding per vagina; the patient was still sick‐looking, afebrile, with moderate pallor and deep jaundice. Her BP was 110/65 mmHg, with PR of 69 beats per minute and random blood sugar (RBS) of 3.4 mmol/L. On per vagina examination, the cervical os was still closed but fresh blood was found on the examining finger. The patient was treated with dextrose, lactulose, and broad‐spectrum antibiotics; after the administration of one dose of Misoprostol, the fetus and the placenta were expelled. Thereafter, the patient was restless, although the vital parameters were in the normal range.

On the 3rd day of hospital stay, the patient became lethargic, with slurred speech and restless, moderately pale, and increasingly jaundiced. Her BP was 119/73 mmHg, with PR of 69 beats per minute, temperature of 36.7°C, and RBS of 4.5 mmoL/L. GCS was 12/15 (E = 4, M = 5, V = 3), with pupils equal and reactive to light, normal tone, and reflexes. The patient was in mild respiratory distress, with a respiratory rate of 28 breaths/minute and oxygen saturation of 95% on room air. The urine was greenish, with a urine output of 300 ml/24 hours. A nasogastric tube was inserted and drained 200 ml of dark brown‐colored fluids. The patient was lethargic and disoriented to time; a diagnosis of hepatic encephalopathy grade 2, based on the West Haven criteria, was made and the plan was to continue the conservative therapy. On the early morning of the 4th day of hospital stay, the patient passed away.

Only after her death, a collateral history could be obtained. The patient's husband, who wanted more children, was not aware that his wife was pregnant. The couple had previously argued on this issue since the patient was not willing to face a new pregnancy, but the husband prohibited her from using any modern contraceptive methods. As reported by the mother‐in‐law, when the patient realized that she was pregnant, she consulted a traditional healer, who provided her a local herb known as “bitter leaf.”

## 3 DISCUSSION AND CONCLUSION

In Uganda, medicinal plants are widely used by indigenous ethnic groups, both as food vegetables and culinary herbs and in traditional medicine. In a study conducted in western Uganda, 75 plants commonly used to induce labor have been identified; most of them possess uterotonic properties and, at unspecified high doses, their toxicity may be life‐threatening for both the mother and the fetus.^14^

**FIGURE 1:**
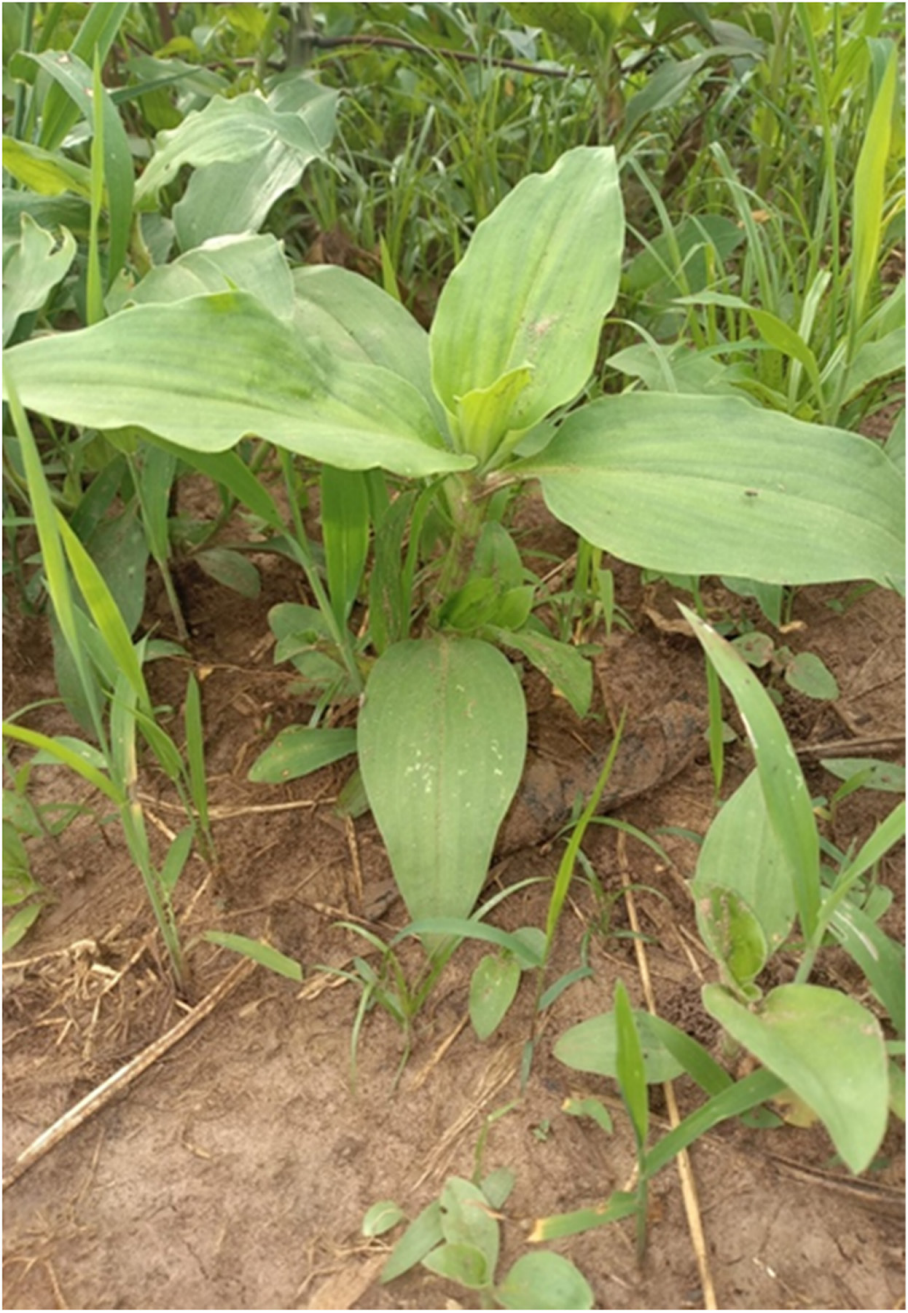
*Commelina Africana* or *“*wandering jew”. Photographs of the herbs that were reportedly used by the two cases following the lead by the traditional herbalists who administered them. The photographs were taken by the authors, CR and GA

**FIGURE 2:**
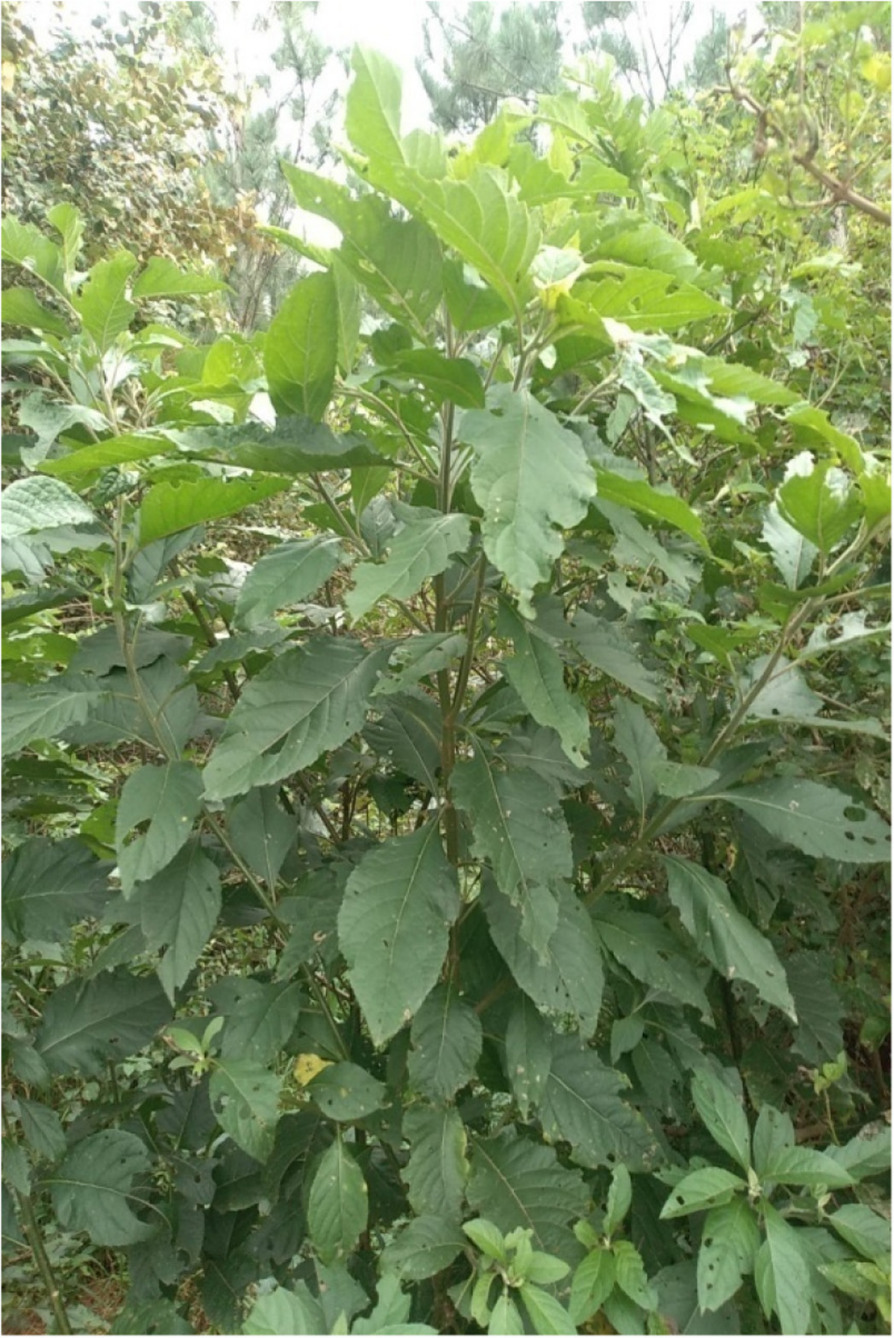
*Vernoniaamygdalina* or “bitter leaf”. Photographs of the herbs that were reportedly used by the two cases following the lead by the traditional herbalists who administered them. The photographs were taken by the authors, CR and GA

In northern Uganda, two plants are commonly used to induce labor, that is, the *Commelina Africana*, also known as *“*wandering jew” (Figure 1), and the *Vernonia amygdalina*, also known as *“bitter leaf”* (Figure 2). The uterotonic properties of these herbs have been already tested and confirmed.^15^ *Commelina Africana* (**“**Wandering jew”; “Lutoto” in the Luo language) is known to have hypoglycemic effects,^10^ as well as uterotonic activity; in fact, it can increase both the force of contractions and their frequency, with a dose–response curve.^15^ To induce abortion, the stem of the plant is normally broken in small pieces and inserted into the vagina, adjacent to the cervix, to induce it to dilate.^14^ The severe complications due to the use of this herb, as reported in Case 1, seem to be due to its insertion in the vagina under unsterile conditions, which allow the ascension of pathogens to the pelvic cavity passing through the cervix, the uterus, and the fallopian tubes, causing massive pelvic abscesses, severe peritonitis, and septic shock. In our case, this condition required multiple surgical interventions with extensive abdominal lavage, as well as the use of reserve antibiotics.

*Vernonia amygdalina* (“Bitter leaf”; “Labwori” in the Luo language) is an indigenous shrub that grows in most parts of sub‐Saharan Africa and may be found growing wild along the edges of agricultural fields. It grows to a height of 1–2 m, with elliptical leaves up to 20 cm long. It is commonly called “bitter leaf” in English because of its bitter taste. As reported by Echemet al.,^16^ *Vernonia amygdalina* plays a big role in the diet of indigenous people throughout equatorial Africa because of the presence of vitamins and mineral salts.^15^ For numerous African ethnic groups, it is also considered a very strong medicine in the treatment of malarial fever, schistosomiasis, amoebic dysentery, and several other intestinal parasites and stomach aches; extracts thereof have been also used traditionally in the treatment of sexually transmitted diseases, cough, constipation, and hypertension.

Its ability to contract the uterus, thereby inducing abortion, has been validated.^15^ *Vernonia* species contain phytochemical principles, which include bitter sesquiterpene lactones, vernolepin, vernodalin, vernomygdin, and steroid glucosides, from which its biological properties were derived.^17^ *Vernonia amygdalina,* especially those grown along heavy traffic routes, can concentrate high levels of heavy metals such as *Iron (Fe)*, *Manganese (Mn)*, *Lead (Pb), chromium (Cr)*, *Zinc (Zn)*, *and Cadmium (Cd)*; which have the potential to act as biological poisons even at parts per billion levels,.^16^ The concentration of these metals is highest in the leaves, which are commonly used for decoctions.

In case 2, the use of *Vernonia amygdalina* led to complications requiring admission to the hospital; the patient presented with reduced GCS due to hepatic encephalopathy, associated with other signs of acute renal and liver failure. Typical laboratory findings were hypoglycemia, leucocytosis, and elevated liver enzymes, associated with elevated creatinine and urea values. The course of the disease was deadly.

In conclusion, we reported two cases describing the unusual severe toxicity of two herbal medicines that are frequently used to induce abortion in northern Uganda. In particular, the use of *Commelina Africana* may be associated with pyometra and/or pyoperitoneum and sepsis, while *Vernonia amygdalina* may lead to acute liver and renal failure, which can be fatal.

The ingestion of medicinal plants to induce abortion involves the risk of severe maternal morbidity and mortality; patients should be informed that the use of these medicinal plants as abortifacients can be life‐treating. Active policy to improve knowledge of contraception and family planning is required.

Moreover, further field and laboratory research is needed to identify the active principles contained in such herbal medicines and their biological properties, to achieve a better understanding of their pathophysiology, dosage levels, and toxicity. This would help in the treatment of the associated complications and the development of possible antidotes.

## AUTHOR CONTRIBUTIONS

CR and GA wrote the case report Background and Discussion; VN collected the information on the patients and compiled the Case Presentation; LS made the final review and editing of the manuscript before submission. All authors have read and approved the manuscript.

## CONFLICT OF INTEREST

The authors declare no conflicts of interest.

## ETHICAL APPROVAL

Ethics approval was provided by the Lacor Hospital Institutional Research Ethics Committee. Before making this case series. Written informed consent was obtained from the patient (Case 1) and the next of kin of the deceased patient (Case 2). The patients' identities were duly concealed.

## CONSENT

Written informed consent for the publication of this case series and the accompanying images was obtained from the patient (Case 1) and the next of kin of the deceased patient (Case 2).

## Data Availability

All data generated or analyzed during this study are included in this manuscript.
